# General Practitioners and Paying Patients

**Published:** 1888-06-30

**Authors:** David Walsh


### General Practitioners and Paying Patients.

By David Walsh, L.R.C.P. and S., Edin.

The Hospitals Association were good enough to invite me to say a few words on the pay system in hospitals, from the standpoint of the general practitioners. First of all, I may say that if anything could have reconciled that long-suffering body to a scheme of the sort, Mr. Burdett-Coutts would have effected it by his essay, which is strong in its tact and sound in its reasoning. At the outset of his paper, the lecturer disclaimed any intention of entering into the question from the medical point of view, but he can hardly be allowed to take up that ground without challenge. The hospitals and the doctors are inseparable, while the application of a principle such as the lecturer advocates would at one blow deprive the profession of a large proportion of its legitimate income. Mr. Burdett-Coutts claims that the question of payment by patients lies at the foundation of the whole hospital system, and it may at once be conceded that if well-to-do patients are admitted to those institutions they should be made to pay to the uttermost farthing in accordance with their means. There is, however, a steadily-growing conviction abroad among the doctors that people who can afford to pay for treatment have no right to accept aid from a charity on any terms whatever: by non-payment they wrong the poor, and by payment they are helping to pauperize the medical profession. If this broad principle can be established, then the foundations of Mr. Burdett-Coutts' splendid structure will be shown to rest upon the sand, and the question is no longer as to the propriety of payment by well-to-do patients, but rather by what right they are in the hospitals at all, and whether their presence is not an active injury to others, who for present purposes may be divided into three classes—First, the really deserving poor; secondly, the subscribers; and, thirdly, the medical profession.

1. *The wrong to the really deserving poor.*—It is obvious that the presence in wards and out-patient departments of those who can afford to pay for treatment must crowd out poor people who would otherwise be there. As things stand, a poor person labouring under serious illness is often sent away from a hospital to get a letter of admission, for which purpose he very likely makes the round of the neighbouring public-houses—a somewhat hazardous tour for a man in robust health. These letters of admission lie at the root of not a little mischief. They render what should be an absolute gift at the disposal of the hospital into a conditional trust, and grant priority of claim to subscribers, however unwise or careless they may be. It is largely owing to such means that improper people gain entrance into wards that were built and endowed for the poor. The admission of paying patients simply means trading on charitable endowments, by the use of beds, house room, and funds meant for other purposes; and the fact that the profits derived from such trading are well applied makes it none the less a misappropriation, and probably, from a technical point of view, an illegal act. Let us imagine one of the wealthy and old-established hospitals to start paying wards which bring in £20,000 a-year. A large proportion of that sum is diverted from the pockets of the medical profession by unfair competition—unfair in the sense that the traders' capital is derived from a charitable endowment, and is worked by the gratuitous services of the medical staff.

2. *The wrong to subscribers*, who, in giving their money to a charity, surely do not intend it for people who can themselves afford to pay for medicine, attendance, and lodging. To put the case in another way, supposing a well-to-do householder were to come to a subscriber and say, "My doctor has ordered me cod liver oil, and quinine, and '47 port, and a course of vapour baths: will you be good enough to pay for these things and find me rooms to live in?" It is a moral certainty that the applicant would at once be shown to the door. But that very same person can go to a public hospital and get at the subscribers' money without let or hindrance. If the door be left open in this way there will always be plenty of people ready to take advantage of it. What does it matter to them that they are defrauding the poor, that they are hoodwinking the benevolent, or that they are evading the just fees due to a humane profession? The modified pay system advocated by Mr. Burdett-Coutts would tax these trespassers, it is true; but then, on the other hand, would it not allow them to claim as a right that which is now taken by stealth? At present, in most cases, there is absolutely no attempt whatever on the part of the hospitals to check such encroachments, and one can hardly wonder at the taunt that interested officials of boards and management seek to swell the rolls of patients for their own self-glorification.

3. *The Wrong to the Profession.*—The hospitals have long ago taken away from the general practitioner patients able to pay small fees, there being no inquiry into the means and fitness of applicants. These small fees must mount up altogether to an immense sum yearly, and now it is proposed, by an extension of the Pay System, to make the robbery more wholesale and sweeping. The hospital authorities, hitherto content with the sprats, are now casting about for the salmon and other more valuable fish, careless of what rights they may be invading. It cannot be too strongly insisted upon that by charging well-to-do hospital patients so far from the wrong to the poor and the subscribers being righted, an additional injury is thrown on the shoulders of a learned profession, whose average income is hardly above that of a middle-class bank clerk. The general practitioner, thus ignored and fleeced by the hospitals, not unnaturally regards those institutions with distrust, suspicion, and indifference.

Let the philanthropist consider a little the position of the general practitioner, who is too often reduced to a condition not far removed from starvation. This state of affairs is in no small measure traceable to the competition of the hospitals, backed up as they are by a charity that is magnificent indeed, but none the less injurious, through a want of reasonable guidance. The general practitioner, with his disunited forces, is a mark for plunder on all hands, and it is in vain he turns for help to the medical corporations, the hospitals, or the medical press. The outside public may contend that the question is to a great extent educational, and that a young man will be a success if he show himself to be worth his salt. That is all very well; but the immediate ordeal is a fiery one, beneath which many are withered and broken. A short time back the statement was circulated that the life of a doctor fresh from graduation is not considered a good policy for the first four years. In other words, it takes five years to kill his ambition, to blunt his susceptibilities, and to ensure his making a living. Let the young man win his spurs by all means; but it is less often a question of honours and distinction than of bread-and-cheese. When the competence is assured the medical man drifts, in most cases, into a mere selfish unit. A little more trades unionism is needed in the profession to look after its interests in lay and other matters.

Without overstating the case, it may be said in conclusion: (1) That there is a strong feeling among the rank and file of the profession that the voluntary Pay System should not be applied in hospitals on any terms whatever; (2) That a great deal of harm may be done on all hands by admitting payments from patients; (3) That strict investigation should be made into the means of applicants by some such machinery as a wage test. If these remarks were to wind up with an abstract amendment, it would be somewhat to the following effect: "That instead of extending the operations of hospitals by adopting a Pay System, it is desirable, by a wise limitation of admissions, to restrict and limit expenditure to its intended object—the relief of the sick poor."

Whatever the immediate result of this discussion may be, every right-minded man must wish to see hospital finance restored to a sound basis, for the subject may be regarded as one of the noblest, and yet the most difficult, of our complex social problems.

